# Identifying genotype specific elevated-risk areas and associated herd risk factors for bovine tuberculosis spread in British cattle

**DOI:** 10.1016/j.epidem.2018.02.004

**Published:** 2018-09

**Authors:** R.J. Orton, M. Deason, P.R. Bessell, D.M. Green, R.R. Kao, L.C.M. Salvador

**Affiliations:** a Boyd Orr Centre for Population and Ecosystem Health, Institute of Biodiversity, Animal Health and Comparative Medicine, College of Medical, Veterinary, and Life Sciences, University of Glasgow, 464 Bearsden Road, Glasgow, G61 1QH, UK; b The Royal (Dick) School of Veterinary Studies, University of Edinburgh, Easter Bush Campus, Midlothian, EH25 9RG, UK; c Roslin Institute, University of Edinburgh, Roslin, Midlothian, EH25 9PS, UK; d Institute of Aquaculture, Faculty of Natural Sciences, University of Stirling, Stirling, Stirlingshire, FK9 4LA, UK

**Keywords:** Bovine tuberculosis, Genotype, Elevated-risk areas, Transitional areas, Herd risk factors

## Abstract

Bovine tuberculosis (bTB) is a chronic zoonosis with major health and economic impact on the cattle industry. Despite extensive control measures in cattle and culling trials in wildlife, the reasons behind the expansion of areas with high incidence of bTB breakdowns in Great Britain remain unexplained. By balancing the importance of cattle movements and local transmission on the observed pattern of cattle outbreaks, we identify areas at elevated risk of infection from specific *Mycobacterium bovis* genotypes. We show that elevated-risk areas (ERAs) were historically more extensive than previously understood, and that cattle movements alone are insufficient for ERA spread, suggesting the involvement of other factors. For all genotypes, we find that, while the absolute risk of infection is higher in ERAs compared to areas with intermittent risk, the statistically significant risk factors are remarkably similar in both, suggesting that these risk factors can be used to identify incipient ERAs before this is indicated by elevated incidence alone. Our findings identify research priorities for understanding bTB dynamics, improving surveillance and guiding management to prevent further ERA expansion.

## Highlights

• Bovine tuberculosis elevated-risk areas historically more extensive than thought.
• Cattle movements alone do not explain spread of areas of elevated risk.
• For all genotypes, the majority of infection spread is due to local effects.
• Similar risk-factors identified within elevated-risk and transitional areas.
• Risk factors key to identify incipient elevated-risk areas before incidence rising.

## Graphical abstract

**Figure:**
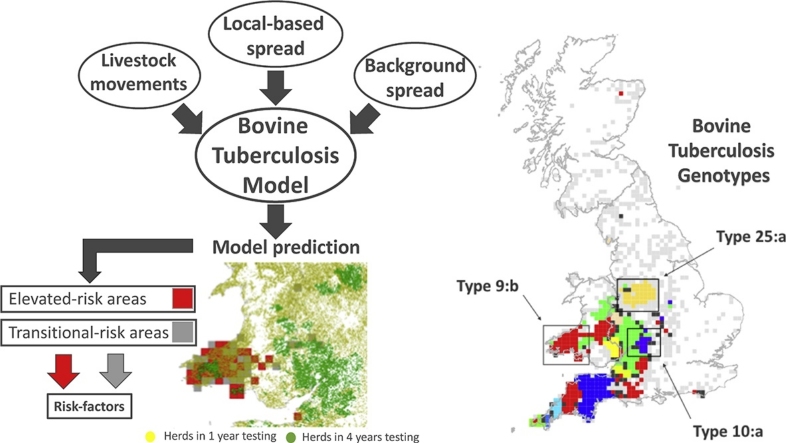

## 1 Introduction

Bovine tuberculosis (bTB) is caused by the pathogen *Mycobacterium bovis (M. bovis)*, and is a disease with important consequences for animal health and production. Historically, bTB has been a major contributor to human TB cases worldwide, and it remains a zoonotic concern in many developed and developing countries (Ayele et al., 2004; Cosivi et al., 1998; Evans et al., 2007). The standard live test used to control bTB in Great Britain (GB) is the Single Intradermal Comparative Cervical Tuberculin (SICCT) skin test, where each animal is checked for an immune response to intradermally injected bTB-derived antigen (de la Rua-Domenech et al., 2006). Control is via a combination of regular test and slaughter using SICCT and abattoir post-mortem testing. Identification of positive test reactors results in a *breakdown*, which places the herd on repeated testing protocols and movement controls until it is deemed clear of infected cattle. In countries that employ a well-developed test and slaughter programme, bTB has either been eradicated (British Veterinary Association, 2009; Radunz, 2006), or has persisted due to the presence of a wildlife reservoir (Nishi et al., 2006; Tweddle and Livingstone, 1994). Both patterns are observed in the British Isles: while Scotland has been declared officially bTB free (British Veterinary Association, 2009), England and Wales have an ongoing bTB epidemic with the Eurasian badger (*Meles meles*) implicated as an important wildlife reservoir for *M. bovis.* This situation is complicated by the protected status of badgers in the UK, and bTB remains a serious and increasing problem in the British cattle industry, with an estimated management cost over £111 m in the 2013/2014 year alone, excluding any Defra policy development costs.^2^ Both the incidence of herd breakdowns and the total area deemed at high-risk of breakdowns increased rapidly in the period after 2001, when foot-and-mouth disease (FMD) resulted in both widespread cessation of routine testing for bTB across GB, and subsequent whole herd restocking of cattle was responsible for widespread dissemination of disease.

The epidemiology of bTB in British cattle has been extensively studied, most notably in the context of the large “Randomised Badger Culling Trial” (RBCT; Bourne et al., 2006; Donnelly et al., 2007; Woodroffe et al., 2006), but also in a number of studies of the respective national epidemics (Brooks-Pollock and Keeling, 2009; Brooks-Pollock et al., 2014; Brooks-Pollock and Wood, 2015; Carrique-Mas et al., 2008; Denny and Wilesmith, 1999; Gilbert et al., 2005; Green et al., 2008; Griffin et al., 1996; Johnston et al., 2005; Karolemeas et al., 2010; Woodroffe et al., 2016). Nevertheless, the expansion of areas in GB with a high incidence of herd breakdowns is still not well understood, and there remains considerable debate over the most appropriate approaches for controlling bTB in cattle (Bennett and Willis, 2007; Conlan et al., 2015; White and Whiting, 2000). Despite the controversy, there is overwhelming evidence of an epidemiological link between bTB in badgers and cattle. First, the culling of badgers is known to be associated with changes in the incidence of herd breakdowns (Donnelly et al., 2006; Griffin et al., 2005). Second, the perturbation of the bTB epidemic caused by the interruption of testing and restocking due to the 2001 FMD epidemic in the UK is similarly correlated to changes in incidence of bTB in badgers (Carrique-Mas et al., 2008). Finally, *M. bovis* genotypes in cattle and badgers are strongly associated at a local geographical level (Woodroffe et al., 2009); this is also consistent with the marked spatial clustering of individual genotypes in high incidence areas (Fig. S1 in Supplementary Information).

Genotyping of *M. bovis* in GB is based on a combination of spoligotyping and variable number tandem repeat (VNTR) typing (Smith and Upton, 2011). *M. bovis* is a member of the *Mycobacterium tuberculosis* complex, which is clonal (Smith et al., 2006), allowing the overall bTB epidemic to be split into multiple discrete genotype-specific epidemics, and therefore one can consider herd breakdowns due to the same genotype as belonging to the same epidemic. Thus, the close association of badger- and cattle-derived genotypes is a strong indicator of transmission between the two species, which can be transmission from badger to cattle or transmission from cattle to badger. All these data are indicative of a single, linked “episystem” with complex interactions at various spatial scales. Identifying the relative roles of the two host species in maintaining and establishing high incidence areas of herd breakdowns is fundamental to improve control in these areas. While we do not know the relative contribution of badgers and cattle to the epidemic, the bidirectional spread between the two species would suggest that the interaction between them is an important part of a disease maintenance system, broadly speaking contained within SW England and Wales, but also responsible for onward transmission of bTB to other areas such as Scotland that would otherwise be without incidents.

In GB, bTB testing was historically managed at the parish level, with herds that were located in high risk parishes of the country tested annually, while those located in low risk parishes tested every two, three or four years according to perceived risk in accordance with criteria listed in European Union directive 64/432/EEC; in recent years, more geographically streamlined designations of High Risk Areas (HRAs) and Low Risk Areas (LRAs) have been introduced. In 2010, all herds in Wales were officially placed on annual routine herd test (The Tuberculosis (Wales) Order, 2010). In England, a new bTB surveillance regime has been in place since 2013, whereby herds in designated LRAs are tested once every four years, herds in HRAs of the west of England are tested annually (DEFRA, 2013), and herds located in a ‘transitional zone’ of intermediate bTB incidence known as the Edge Area (EA) are tested annually. Herds that are located in increasing bTB incidence parts of the EA are tested every 6 months since January 2015. However, as of late 2017, the testing regime in EA has been under review (Animal and Plant Health Agency, 2017).

Breakdowns can potentially be seeded a considerable time prior to detection. As test intervals have historically been at least in part determined by the local breakdown incidence, in a spatially expanding epidemic, testing could lag behind the establishment of new areas where cattle herds are at a higher risk of a breakdown. In this case, this could also be associated with interaction with reservoir hosts. A critical component to understanding how areas with elevated risk of bTB spread, and therefore how to best control them, is the development of epidemiologically driven definitions of these areas. In this analysis, we propose a novel approach to identifying areas with elevated risk for three geographically discrete bTB genotypes, utilising the predicted impact of recorded cattle movements to estimate the role of unobserved transmission in a likelihood based-setting, in conjunction with the recorded spatial distribution of *M. bovis* genotypes. To avoid confusion with the already established formal term High Risk Areas (HRAs), we will designate our estimated high-risk areas as elevated-risk areas (ERAs). We then determine the total probability of infection due to three factors: (i) livestock movements, (ii) local-based spread, and (iii) a background, country-wide rate. Finally, we test for any significant differences in risk factors between the identified ERAs and the transitional areas (TAs) (areas with intermittent elevated risk during the study period), as well as assess any general trends of spatial spread of bTB in England and Wales. In this analysis, we concentrate on the years 2002–2008, a period when the rapid expansion of bTB meant that the signature of transition is likely to be most marked.

## 2 Materials and methods

Breakdowns are often detected only after harbouring infection for a considerable time (Karolemeas et al., 2011). This is exacerbated by the differences in testing regimes across GB. To identify areas likely to harbour hidden infections, we examine the “shadow” of breakdowns caused by outward cattle movements from herds at higher risk of having undetected infected animals. A previously published model (Green et al., 2008) used this concept to estimate the relative proportion of transmission due to movement-based spread, using the explicit dynamic social network that is defined by recorded cattle movements. Green and colleagues (Green et al., 2008) used parishes under one- or two-year testing as a proxy for ERAs or, alternatively, ERAs were defined by 6 km circles centred around breakdowns from the previous year. Here, we adapt this approach to explicitly identify putative ERAs for specific genotypes utilising a novel grid-square approach.

### 2.1 Source data

Cattle movements were extracted from the Cattle Tracing System (CTS) of GB (provided by RADAR), and bTB breakdown details were extracted from the animal health database VetNet (provided by Defra). The model considered 136,302 premises identified by the CTS that have had at least one recorded movement, where the data had been cleaned and premises coordinates were available. The model utilises the movement of all cattle, which are represented as daily links between pairs of premises. Movements to slaughter were removed. As markets involve transient contact at best between cattle, stays at markets were not considered as infectious (Skuce et al., 2011), and were removed from the dataset such that movements A → B → C, where B is a market, were replaced by a single movement A → C set to occur on the recorded date of arrival at C. Individual movements with equal dates, start, and end points were grouped into batches. For 2002–2008, there were 6,625,056 resulting batch movements with a mean batch size of 3 animals.

Here, we consider only breakdowns confirmed by successful culture of *M. bovis*, as this is also a requirement for obtaining bacterial genotypes. For 2002–2008, there were 15,939 such confirmed breakdowns. Of these, 99.4% were matchable to unique county/parish/holding (CPH) codes present in the CTS data, across 10,838 different premises.

Genotype data consisting of spoligotypes and VNTR types were obtained from the Animal and Plant Health Agency (APHA). Three genotypes were chosen for investigation on the basis of their geographical predominance in expanding regions of high incidence, at the edge of annual testing areas. As defined by the international naming convention (Smith and Upton, 2011), these were genotype 25:a (spoligotype 25 [SB0129], VNTR type 6-5-5-4*-2-3.1, mainly prevalent in the English West Midlands), genotype 10:a (spoligotype 10 [SB0272], VNTR type 7-5-5-4*-3-3.1 on the Welsh border) and genotype 9:b (spoligotype 9 [SB0140], VNTR type 7-5-5-5*-3-2.1, in South West Wales). Each of the three genotype datasets were linked to breakdowns in VetNet via cattle eartag numbers to generate a list of herd breakdowns for each genotype. For 2002–2008 there were 1134 breakdowns for genotype 25:a, 465 for genotype 10:a, and 1091 for 9:b.

### 2.2 Individual Premises Model

#### 2.2.1 Model construction

The model operates at the individual premises level. Each premises *i* maintains a probability of infection through the simulation, *P_i_*, incremented using one-day timesteps. Each potential infection event causes infection with probability *p*, which causes an increase in *P_i_*, conditional on the probability of *i* already being infected, such that

$$P_{i}\mapsto P_{i}+\left(1-P_{i}\right)p\;.$$

The summation of $\left(1-P_{i}\right)p$ across all infection events of all types gives the expectation of the total number of infections produced during the simulation. The model partitions this into three infection types which record the total probability of infection due to: (i) livestock movements, (ii) local-based spread, and (iii) a background, countrywide rate. The expected prevalence at a given time is given by $\underset{i}{\sum }P_{i}$.

The value of *p* depends on the particular type of event that occurs:

i) Livestock movements. The movement of an animal from a premises is considered to be infectious in proportion to a probability *μ *per animal moved. We assume that the probabilities that single animals are infectious are independent of each other, and therefore, a batch movement of multiple animals from premises *j* to premises *i* carries with it a probability of infection

$$p=\left(1-{\left(1-\mu \right)}^{c}\right)P_{j,}$$

where *c* is the number of cattle in the movement batch. Herds in ERAs will usually have a much higher probability *P*, and thus onward movements of cattle from these herds will in turn contribute to more infection in herds that receive cattle.

ii) Local-based spread. Within model-defined ERAs, we assume that all cattle herds are at a more elevated risk of infection due to local processes that are independent of recorded cattle movements; this is also termed local-based spread. We do not consider in detail within-premises infection, or heterogeneity amongst premises in the risk of infection through this mechanism, nor do we explicity consider the effect of a wildlife resevoir. Therefore, we assume all premises within these areas are subject to infection with constant daily rate *γ*.

iii) Background rate. Every premises is considered to be exposed to infection on a daily basis with a small fixed rate *β*, independent of location or movement; the model considers a daily infection event  *β* for each premises. This rate simulates infection due to unknown causes that are not included in the other model processes, such as spread via unrecorded cattle movements or contacts, or spread from an unobserved reservoir population.

#### 2.2.2 Model evaluation

All of GB was divided into 10 × 10 km quadrats, with quadrats designated at elevated or lower risk (see Identification of ERAs below). Model simulation fits were run over a three-year time window, consisting of three different year-long stages: the pre-seed, seed, and test stages. Breakdowns from the seed year in the selected elevated-risk (ER) quadrats were used to set the index cases that define the initial state of the model. For each index case breakdown *i*, occurring at time *t^brk^*, premises *i* was considered infectious over time {*t^brk^ − w*, *t^brk^*} with * *P_i_ *= 1. Parameter *w,* therefore, represents the time elapsed between a premises being infectious and its eventual detection. Parameter *w* is set to 365 days, hence, index cases are infectious during periods of both the pre-seed and seed years. The model’s predictions for the final year stage − the test stage − were then tested against the VetNet breakdown data for that year. The variable *Y_i_* represents the premises infection status inferred from the breakdown data. For all herds that had a breakdown in the test year stage, over the window {*t^brk^ − w, t^brk^*}, otherwise ** *Y*_*i*_ = 1.

Where {*Y*} is the set of all premises, model likelihood was calculated as a product over the probabilities assigned to all herds in the evaluation window:

$$L=\underset{i\in Y}{\prod }P_{i}^{Y_{i}}{\left(1-P_{i}\right)}^{\left(1-Y_{i}\right)}\text{.}$$

Additionally for breakdown premises, *P* was set to 0 on day *t*^*brk*^ + 1 to account for removal of test reactors and imposition of movement restrictions.

#### 2.2.3 Parameter fitting

An adjustment to *P* was made before calculating *L* to account for rounding errors leading to unit probabilities for a small number of premises, which would lead to undesirable zero likelihood statistics. Therefore, we set

$$P_{i}^{\text{'}}=\frac{P_{i}+\delta }{1+2\delta },\delta =10^{-10}\text{.}$$

The value of δ is arbitrary. Goodness of fit of the model was then expressed in terms of the log-likelihood. The Downhill Simplex or Nelder-Mead (NM) algorithm (Nelder and Mead, 1965) was used to find the maximum likelihood solution, fitting model parameters *β*, *μ*, and *γ*. Since the algorithm is inherently unbounded, boundaries were imposed on the model parameters using a logistic function:

$$x\text{'}=\frac{x_{max}}{1+e^{-x}},x\in \left(-\infty ,\infty \right);x\text{'}\in \left(0,x_{max}\right),$$

where parameter *x* is fitted but mapped into parameter *x*' for use in the model, with range (0, *x_max_*).

#### 2.2.4 Identification of ERAs

A three-phase process was used to identify ERAs. In the first phase, each quadrat for a specific genotype was used in a separate simulation as the sole ERA for that genotype. Only premises located inside the ERA are deemed at high-risk of infection and subjected to daily local spread *γ* and only breakdowns of the selected genotype located inside the selected quadrat are used as index case seeds. Any quadrat with two or more breakdown premises of a specific genotype during the period 2002–2008 was considered for evaluation as an ERA. For genotypes 25:a, 10:a, and 9:b this resulted in 48, 46, and 64 quadrats for evaluation, respectively. Epidemic likelihoods were obtained from five independent NM simplex fits, with the highest likelihood observed used to rank the quadrats. In the second phase of the model selection, these quadrats were then ranked and sequentially aggregated into a larger ERA (which is not necessarily continuous), from highest to lowest likelihood as identified in the previous phase. The model likelihood was re-evaluated and parameters *β*, *μ*, and *γ* refitted after each quadrat was sequentially added. For any given year, a maximum likelihood value will be observed at a specific number of quadrats (N) that therefore represents a first-order approximation of the ERAs in that year. In the third phase, this approximation was then improved by reordering the quadrats, so that those that resulted in a reduction in likelihood upon their aggregation into the ERA were re-ranked to after the quadrat with the maximum likelihood. For example, an initial ordering of A, B, C, D, E where the aggregation of B actually resulted in a lowering of the likelihood in phase two, whilst the incorporation of D gave the overall maximum likelihood, would be reordered to A, C, D, B, E. The model was then re-run with these re-ranked quadrats which were again sequentially aggregated into a larger ERA, and the model likelihood was re-evaluated with parameters *β*, *μ*, and *γ* refitted each time. The final ERA extent was then defined by the model output as the aggregated ER-quadrats that gave the maximum likelihood. This process was repeated for five different three-year evaluation windows, with the years 2003–2007 used as different seed years, resulting in a picture of ERA spread for 2004 up to 2008 for each genotype.

### 2.3 Risk factor analyses

These analyses tested for any significant differences in risk factors for bTB infection associated with 1) different genotypes and 2) areas of varied bTB risk. A bTB risk area was determined by analysing the persistence of risk of ER-quadrats across years. We considered an ER quadrat an ERA if it was designated ER by the model in all 5 years, and a transitional area (TA) if it was intermittently designated ER during the 5 years and ER in the last year.

#### 2.3.1 Multivariate Logistic Mixed Models

Individual multivariate logistic mixed models without interactions were fitted for each studied genotype to risk factors previously associated with the infection of bTB (Bessell et al., 2012). The binary model response variable was either 1 if a premises recorded any confirmed bTB incidence of that genotype for a given year, or 0 otherwise. Breakdowns from other genotypic strains were also coded as 0. Year acted as a random effect to estimate the annual mean distribution of bTB prevalence and to control for possible fluctuations on the annual number of breakdowns. Finally, data from each genotype were combined to assess any general differences between ERAs and TAs. The following fixed effects were fitted:

1. Herd type: The different herd types recorded in VetNet were consolidated into five groups: beef, dairy, suckler, finishing and store. Other herd types were excluded from the analysis because of records' ambiguity (e.g. “mixed”, “calf rearer”) or incomplete information (e.g. “other”, “not known”).
2. Herd size: The annual mean herd size (divided by 100) was calculated using CTS data.
3. Cattle movements: The (squared-root of the) annual number of inward batched cattle movements received by premises from the 100 km^2^ quadrats deemed to be at an elevated risk for a particular genotype.

The data were prepared using AWK (Aho et al., 1987) for text processing and extraction, and R for data management, model fitting and model assessment. The multivariate logistic mixed models were constructed in the lme4 package (Bates et al., 2014) in R (R Core Team, 2017).

Additional genotype-specific models were fitted using area risk type (either ERA or TA) to create an interaction term for each risk factor. These models were then used to calculate pairwise contrasts of least square means to summarise the effects of the different herd types in each area, within each genotype-specific model. These calculations were performed with the lsmeans package (Lenth, 2016) in R.

#### 2.3.2 Model assessment

Receiver Operating Characteristic curves (ROC curves) were plotted to illustrate the accuracy of each model. True- and false-positive rates, as well as the area under the curve (AUC) were calculated with the ROCR package (Sing et al., 2005) in R.

## 3 Results

**Fig. 1:**
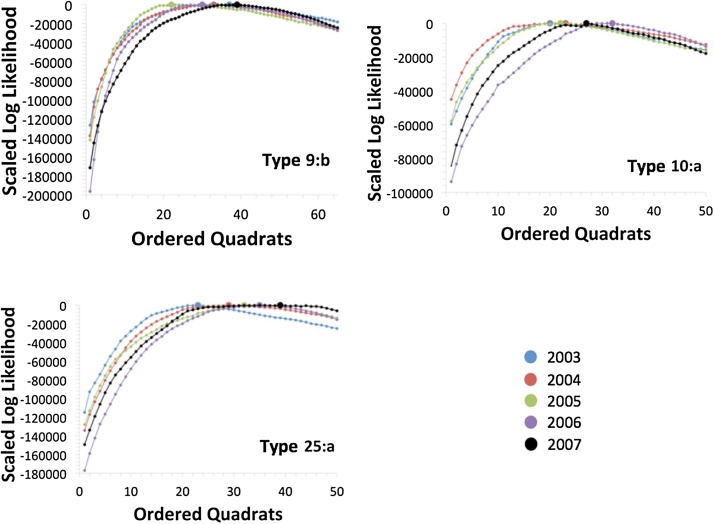
Aggregate likelihood statistics for genotypes 9:b, 10:a, and 25:a, where the large circle represents the maximum likelihood for that genotype–year combination. Years represented are 2003 (blue), 2004 (red), 2005 (green), 2006 (purple), and 2007 (black). (For interpretation of the references to colour in this figure legend, the reader is referred to the web version of this article.)

The sequential aggregation of ranked quadrats into the putative ERAs initially resulted in increased model likelihood (Fig. 1), though with some small fluctuations. Beyond the maximum likelihood value, likelihoods typically declined at a lower rate per quadrat, compared to the initial increase in likelihood up to the maximum. This would imply that, while core ERAs are relatively well defined, there is greater uncertainty in identifying which areas are not ERAs, possibly because these usually contain fewer breakdowns due to an inherent lower incidence or because these are typically in quadrennial testing areas.

**Fig. 2:**
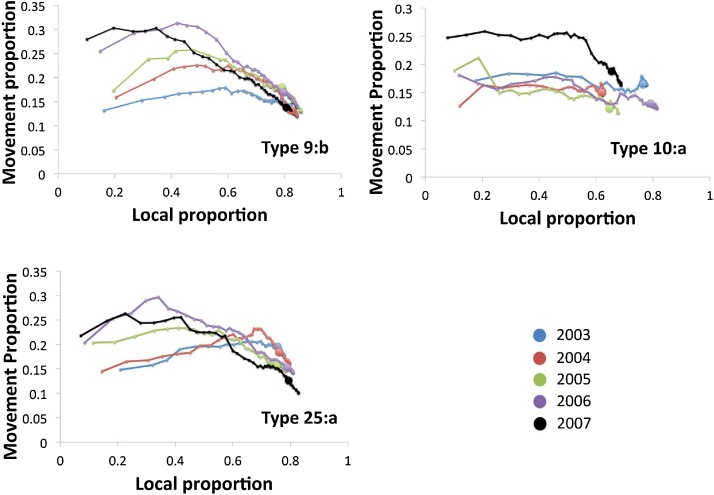
Scatter plots, showing changes in the proportion of breakdowns explained by recorded cattle movements and local effects. An individual series represents the proportion of breakdowns attributed to movements and local effects at each stage of the quadrat aggregation of the ERA for a given genotype in a given year; for genotypes 9:b, 10:a, and 25:a in years 2003 (blue), 2004 (red), 2005 (green), 2006 (purple) and 2007 (black). A large circle represents the maximum likelihood ERA aggregation for each series. Attribution of breakdowns to movements initially increases, but decreases as the ERA aggregates further to the maximum likelihood solution, attributing the majority of breakdowns to local effects. (For interpretation of the references to colour in this figure legend, the reader is referred to the web version of this article.)

The majority of the change in the attribution of breakdown sources as more quadrats are included is due to an increase in the number of breakdowns caused by the local parameter at the expense of background. However, the decrease in the proportion of breakdowns attributed to movements as the area encompassed by ERAs increases also suggests that, at the local level, there is a considerable trade-off between breakdowns attributed to movements, and those attributed to local-based spread (Fig. 2).

The cattle movement network is extremely dense, and thus the maximum proportion of the epidemic that can be explained by movements can potentially be high: this is up to 30% in the case of genotype 9:b in 2006, at the point when the estimated ERA encompasses only the five highest ranked quadrats in 2006 (Fig. 1). However, this model is not the optimal one, and the best-fit model in all cases suggests a lower contribution of movements to the epidemic: the optimal ERA for genotype 9:b in 2006 encompasses 30 quadrats (Fig. 1) with movements contributing 15% (Fig. 2). The rapid initial rise in the role of movements as ER quadrats are accumulated is soon reversed, as the estimated ERAs expand to the maximum likelihood solution and encompass increasing numbers of breakdowns. The short-range, contiguous accumulation of ERAs also suggests that cattle movements are unlikely to be the explanation for the majority of ERAs spread, as they are typically much farther than the 10 km quadrat length scale, with a median distance of just under 58 km (Mitchell et al., 2005).

**Fig. 3:**
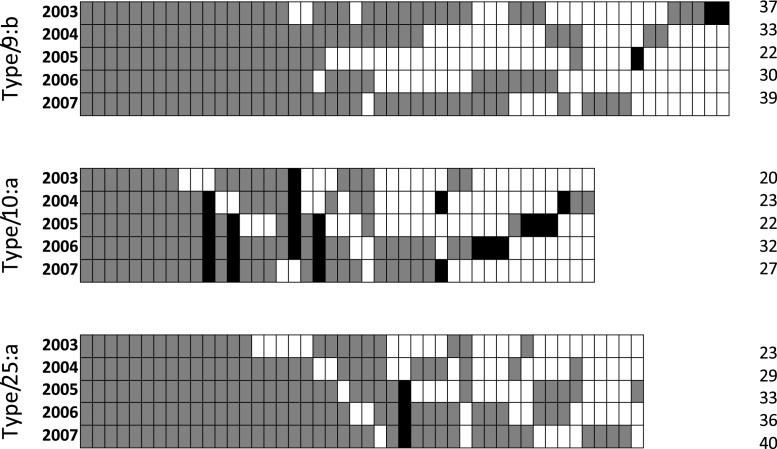
All elevated-risk quadrats for the three genotypes, showing years in which they are designated at elevated-risk by the model. Quadrats that appear within the largest contiguous group are in grey, those that appear in isolation (i.e. outside the largest group) in black. Quadrats are ordered from left to right in terms of the number of years of consistent designation as an ERA.

**Fig. 4:**
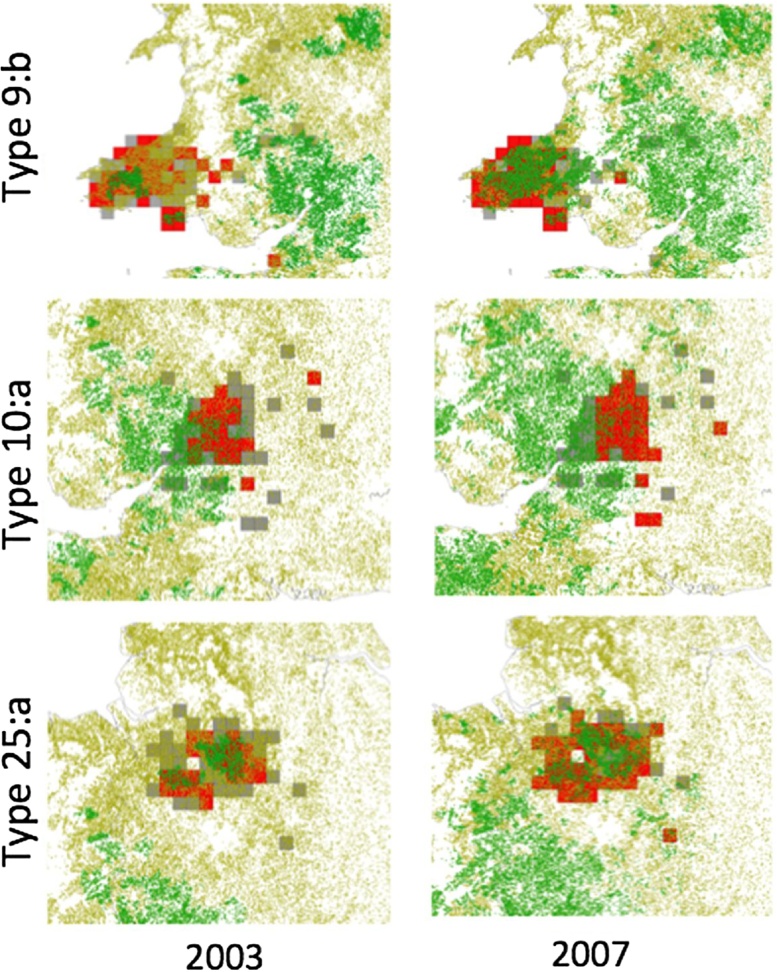
Geographical spread of Elevated-Risk Areas for types 9:b, 10:a, and 25:a in 2003 (left) and 2007 (right). Model-predicted elevated-risk quadrats for the given year are shown with red squares, whilst those evaluated by the model (as two or more recorded breakdowns of the specific genotype) but not deemed at elevated-risk are shown with grey squares. Whilst, the green dots represent herds under annual and biennial testing, whilst yellow dots represent herds under 3 or 4 year testing. (For interpretation of the references to colour in this figure legend, the reader is referred to the web version of this article.)

Of the three genotypes, type 25:a shows clear signs of expansion over the years 2003–2007, whilst type 10:a shows a less consistent expansion, and type 9:b an apparent contraction before re-expansion (Fig. 3 and Figs. S2-S4 in Supplementary Information). However, all 3 types show a clear “core” ERA encompassing contiguous quadrats that are always at elevated-risk, as well as contiguous quadrats that are often (but not always) at elevated-risk (Fig. 3). Furthermore, the model predicts quadrats to be at elevated-risk well in advance of the herds within them becoming officially high risk (i.e. annual or biennial testing) (Fig. 4, Table 1). This suggests that the model predicted ERAs are not only robust, but that the approach is capable of identifying potential elevated-risk regions well in advance of perceived risk, at least as identified at the time.

**Table 1:** Percentage of herds in the model-predicted Elevated-Risk Areas that were under annual or biennial testing by seed year (2003) to (2007).

Year	% of Model ERAs herds in annual testing areas		
	Genotype 9:b	Genotype 10:a	Genotype 25:a
**2003**	20.4	66.1	50.1
**2004**	47.1	61.9	41.7
**2005**	73.5	60.9	61.5
**2006**	75.4	63.0	55.6
**2007**	75.2	56.7	48.9

Analysed over the five years, the best-fit linear growth rates for the area estimated as ERAs are low, with only type 25:a showing a significant non-zero increase (320 km^2^ per year, R^2^=0.98). This overall slow annual growth rate is consistent with local, contiguous spread at the analysed 10 km scale. Not only is the establishment of new ERAs largely short range, but identified ERAs were considerably in advance of where annual testing parishes were being established, most markedly in the case of genotype 9:b, where in 2003, fewer than 21% of ERA herds are annually tested (Table 1).

**Fig. 5:**
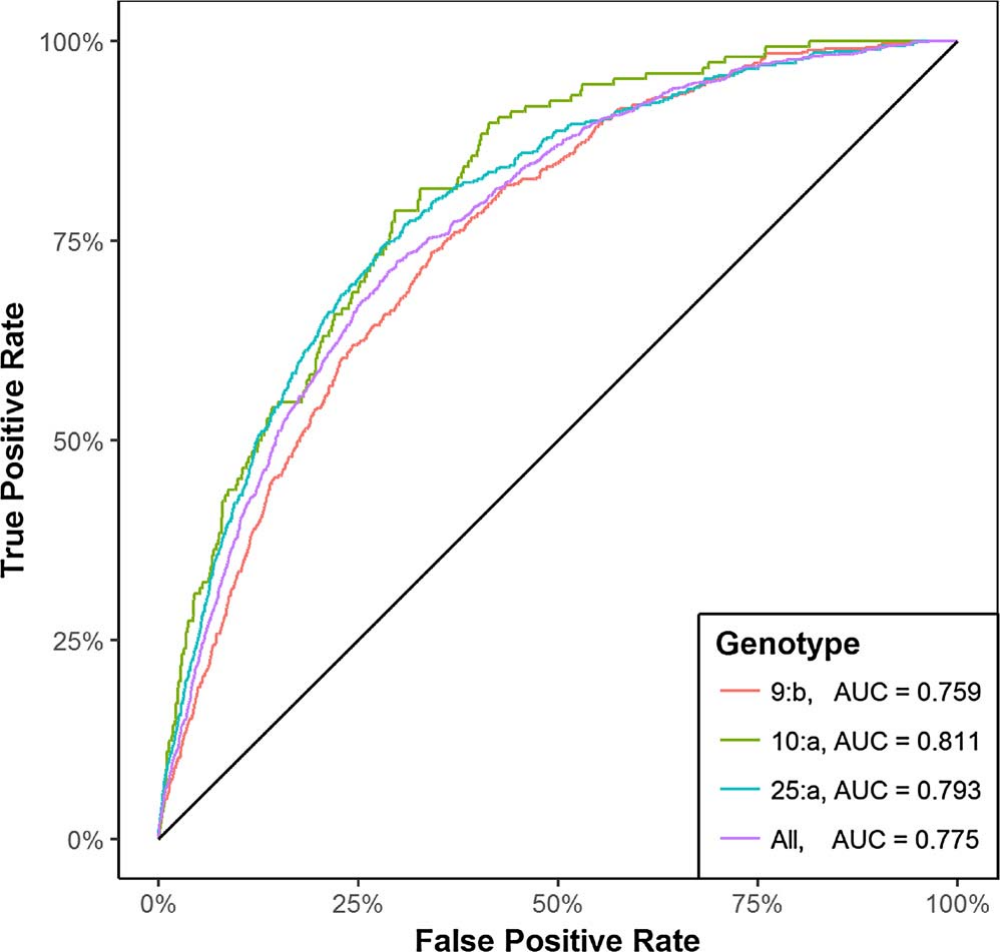
Receiver operating characteristics curve for the models fit for the three genotypes of interest and the combined data (“All”). The area under the curve (AUC) is presented next to each respective model in the legend. The solid black line represents an uninformative model equivalent to assigning the binary response at random. (For interpretation of the references to colour in this figure legend, the reader is referred to the web version of this article.)

The genotype-specific models developed to compare the premises’ risk of a bTB infection have very good classification ability, as indicated by the AUC values (Fig. 5). The odds ratios with 95% confidence intervals and the number of genotype-specific breakdowns by genotype are presented in Table 2. ERAs, herd size, and ER movements were significant in each model. The analysis shows that premises within an ERA are 4.48, 5.75, 4.36 and 4.38 times riskier than premises within TAs, for genotypes 9:b, 10:a, 25:a and the combined genotypes, respectively.

**Table 2:** Model output for the three genotype-specific models as well as the combined genotype model. Odds ratios are presented with 95% confidence intervals. Asterisks denote varying levels of statistical significance (^*^p < 0.05; ^**^p < 0.01; ^***^p < 0.001).

	Genotype 9:b	Genotype 10:a	Genotype 25:a	Combined Genotypes
Transitional Area [reference level]	1	1	1	1
Elevated-risk Area	4.48*** (3.49, 5.74)	5.75*** (3.94, 8.38)	4.36*** (3.49, 5.46)	4.38*** (3.77, 5.09)

*herd type*				
Beef [reference level]	1	1	1	1
Dairy	0.77 (0.41, 1.44)	1.14 (0.68, 1.90)	2.19*** (1.53, 3.12)	1.36* (1.08, 1.72)
Finishing	0.71 (0.32, 1.55)	1.32 (0.79, 2.22)	1.12 (0.71, 1.78)	1.09 (0.80, 1.49)
Store	0.58 (0.25, 1.36)	1.25 (0.57, 2.72)	1.23 (0.69, 2.20)	1.03 (0.70, 1.53)
Suckler	0.53 (0.28, 1.01)	1.16 (0.71, 1.88)	1.45 (0.97, 2.17)	1.04 (0.81, 1.33)

Mean annual herd size/100	1.30*** (1.23, 1.37)	1.44*** (1.32, 1.58)	1.38*** (1.31, 1.45)	1.37*** (1.32, 1.41)
Elevated-risk movement	1.01** (1.00, 1.02)	1.10** (1.03, 1.18)	1.01* (1.00, 1.03)	1.01** (1.00, 1.02)

*intercept*	0.01*** (0.01, 0.03)	0.01*** (0.01, 0.02)	0.005*** (0.003, 0.01)	0.01*** (0.01, 0.01)
Observations	11,115	2762	13,067	26,944

The risk associated with herd size was highly significant [p < 0.001] and very similar between the different genotype groups, generating odds ratios of 1.30, 95% CI [1.23, 1.37], 1.44, 95% CI [1.32, 1.58], 1.38, 95% CI [1.31, 1.45] and 1.37, 95% CI [1.32, 1.41] for genotypes 9:b, 10:a, 25:a and the combined genotypes, respectively. This can be interpreted as every additional 100 cattle to the mean herd size being associated with a 30–44% increased risk in a breakdown. The risk associated with an elevated-risk movement was the highest in genotype 10:a (with odds ratio of 1.10, 95% CI [1.03, 1.118], p < 0.01, compared with 1.01, 95% CI [1.00, 1.02], p < 0.01, for genotype 9:b, and combined genotypes, and 1.01, 95% CI [1.00, 1.03], p < 0.05, for genotype 25:a), meaning that each additional batch of cattle from a particular genotype region was associated with a 10% increase in risk for genotype 10:a. There was only a nominal 1% increase in risk for the remaining genotypes. Additionally, dairy herds were shown to be 2.19, 95% CI [1.53, 3.12], p < 0.001, times riskier than beef herds in genotype 25:b, while dairy herds in the combined genotype model were shown to be 1.36, 95% CI [1.08, 1.72], p < 0.05, times riskier than beef herds. The risk associated with herd type was not found to differ significantly between beef herds and the remaining herd types for genotypes 9:b and 10:a.

Restructuring the previously fitted genotype-specific models with interactions between area type and herd type permitted the risk of each herd type in the different risk areas to be evaluated. Model-specific mean values of annual herd size and ER movements were used when calculating the pairwise model contrasts. The genotype-specific risk factor models and pairwise contrast models have identical fitting values (with identical model deviances). The estimated differences in least squares means with standard errors, standardised z-scores and *p*-values for each pairwise contrast by herd type and genotype-specific risk area are presented in Table 3. In all models, premises in TAs were less likely to contract bTB of a specific genotype than in ERAs.

**Table 3:** Area type pairwise contrast of least squares means for each genotype and for the combination of the three genotypes. Differences in risk for Elevated-Risk-Areas are shown, using transitional areas as a reference. Odds ratios with 95% confidence intervals are shown. Asterisks denote varying levels of statistical significance (^*^p < 0.05; ^**^p < 0.01; ^***^p < 0.001).

	Genotype 9:b	Genotype 10:a	Genotype 25:a	Combined Genotypes
Beef	6.4*** (2.9–14.12)	3.8** (1.81–7.98)	2.07 (0.61–6.69)	3.79*** (2.36–6.07)
Dairy	4.9** (2.08–11.57)	4.74*** (3.35–6.7)	5.16*** (3.43–7.77)	4.75*** (3.71–6.09)
Finishing	3.75** (1.63–8.64)	4.82** (2.08–11.14)	3.69* (1.21–11.28)	3.9*** (2.34–6.48)
Store	4.12* (1.04–16.28)	3.62* (1.26–10.42)	1.28 (0.4–4.11)	2.43** (1.25–4.72)
Suckler	9.64*** (4.26–21.79)	3.78*** (2.16–6.63]	6.73*** (4.05–11.17)	5.47*** (3.93–7.61)

## 4 Discussion

In this paper, we have exploited the spatial clustering of *M. bovis* genotypes to gain insight into which specific geographical regions have an elevated risk of bTB spread. Using this combination of spatial, network and genotype information, we have developed novel definitions for areas where British cattle are at an elevated risk of becoming infected with *M. bovis*. By using the pattern of outbreaks, we are able to disentangle the relative contributions of movement and other factors within Elevated-Risk Areas (ERAs). Such an approach provides a better definition than attribution based on incidence of breakdowns, especially when these differences are confounded by the combination of different testing regimes in use. The pattern of undetected exposures could affect our analysis if they are meaningfully biased when compared to the pattern of reactors, although it is more likely that these missing data simply imply greater uncertainty in the attributed ERAs, with delays in identifying their emergence as the number of confirmed reactors increases. Thus our conclusion, that ERAs appear long in advance of apparent spread, should be robust.

The steep rise in likelihood to the maximum value as quadrats are accumulated would suggest the areas designated at elevated risk are usually robustly defined, due to their large contribution to the increase in likelihood. However, because of the shallow decline in likelihood thereafter, the total ERA size may be sensitive to a reordering of the quadrats. Although our search algorithm for assigning ERAs includes two rounds of optimization, it is not guaranteed to find a globally optimal solution. The optimal ordering of quadrat accumulation might produce even larger ERAs for any given year, though a few quadrats would also be re-designated as non-ERAs. The slow decrease in likelihood beyond the minimum also highlights one of the key difficulties in separating movement-based and local-based transmission involved in studies within ERAs, such as the RBCT: cattle movements are frequent and the network of contacts dense, so many breakdown incidents could be attributed to movements even though other factors may be more important. Utilising the data on spread outside ERAs avoids this problem due to the absence of other likely mechanisms of long distance spread.

We also note that historical differences in the control policies between England and Wales, such as ‘The Tuberculosis (Wales) Order 2006′ and ’The Tuberculosis (England) Order 2007′ could account for some of the differences in results between genotypes. In particular, policies such as pre-movement cattle testing implemented in Wales in 2006 could have influenced the elevated-risk area for genotype 9b model, as the designation of a quadrat being elevated-risk is in part related to the number of breakdowns and, therefore, tests in each quadrat. However, a similar policy in England in 2007 did not seem to unduly influence our results in the last year of the study.

Further refinements to the model might include an explicit weighting of ERAs by their apparent contribution to the epidemic, rather than the binary elevated/lower risk approach used here. For example, a region deemed at lower-risk for four years could have a higher weighting than one that is designated at elevated-risk for a shorter period. In addition, more sophisticated fitting procedures are possible. We use the likelihood values as a simple measure for model selection but reverse jump MCMC, for example (Green, 1995; Streftaris and Gibson, 2004), would allow us to evaluate the possible independent role of each quadrat as an ERA. However, the potential for greater resolution is counterbalanced by the considerable cost in computational effort. Indeed, given the size of the cattle movement dataset it is likely that such an approach would require a greater abstraction in either the underlying model or the data being used. Furthermore, prior versions of the model were tested for robustness under longer timescales, with dynamic ERAs, different infectious and test windows (from 70 to 365 days), higher susceptibility associated with breakdown premises (compared to all others), and increased susceptibility of larger farms. None were found to provide meaningfully better model results (Green et al., 2008). A further model enhancement would be to allow variation in the probability of infection due to local spread between ERA quadrats. This probability could potentially be informed by agricultural, ecological, and geographical parameters such as cattle/badger density and land type/barriers.

New ERAs tend to appear contiguously to existing areas, with only relatively rare appearances of ERA quadrats in isolated locations. These isolated ERAs are typically transient (Fig. 3) and are associated with multiple short-term outbreaks. These were classified as Transitional Areas (TAs) while ERAs were considered at elevated-risk for the entire study period. Risk factor analysis showed that premises in TAs were less likely to contract bTB than in ERAs for all genotypes, which supports the simulation model’s ERA assignments. While the odds ratios associated with risk factors in ERAs are considerably higher than in TAs, nevertheless the risk factors themselves are consistent across both area types. Importantly, these are also substantially different from risk factors associated with low incidence areas (Bessell et al., 2012; Salvador et al., 2018), suggesting a signature that could be associated with incipient ERAs.

The pattern of spread we have identified generates insights into the bTB epidemic in GB at the national scale. The spread of ERAs could be due to cattle movements, local cattle-related activities, environmental contamination, badger-to-badger spread, or a combination of these. Although cattle movements are important, they are not attributed to be the primary source of infection in our models. Further, considerable outbreaks in cattle have been observed in naive regions, but these do not result in persistent ERAs (Gopal et al., 2006), and thus establishment of ERAs due to cattle involvement alone would seem to be unlikely. Additionally, the locations of new ERAs are not well described by the distribution of cattle movements from ERAs. This would imply that any attempt to control spread of ERAs would require barriers far in advance of the apparent limits of high breakdown incidence. While our model in itself does not allow different local effects (badger or cattle) to be quantified directly, badger-to-badger spread is on the whole more consistent with the picture observed here, as badgers are largely sedentary and stick to a local territory. Some reports suggest using badger culling or vaccination as a ‘firebreak’ to prevent any further ERA spread, and this work can therefore help to identify where such firebreaks should be placed.

In recent years, all cattle herds in Wales and a substantial part of England have been placed on annual testing schedules, and some herds are tested every 6 months in areas of increasing incidence. As these measures were put in place so as to test more frequently in advance of these regions becoming ERAs, it is likely that the gap we observe between attainment of elevated risk and ascertainment of it, would become shortened. We would still propose, however, that this approach could remain a useful one to identifying areas that are at the greatest risk of infection, and those areas that are not.

In summary, while there remain many important questions regarding the spread of bTB in British cattle, our analysis provides a basis for refining further comparisons of lower- and elevated-risk areas, and the reasons for their transition. The disparity between the historical testing regime and our attributions of ERAs suggest that such differences may be important both in terms of refining epidemiological analyses, establishing the best means of controlling the spread of bTB, and identifying early warning signatures of ERA establishment. Furthermore, our approach can be used to identify properties of other management–reservoir systems, especially where well-defined contact structures can be identified in the management host. While we use the extremely explicit contact structure available for British cattle, the approach does not require such detail, and could be applied, for example, using simpler or lower-resolution models of contact.

## Data accessibility

Data from CTS database were supplied by the RADAR Unit at Defra and data from the Vetnet and genotype databases were supplied by APHA. These data cannot be made freely available to the general public due to legal restrictions but application for access can be made by direct application to Defra.

## Competing interests

The authors have no competing interests.

## Author contributions

This project was conceived by RRK. The modeling framework and analysis were designed by RRK, RJO, and DMG, and implemented by RJO. The genotype risk factor analysis was designed by RRK, LCMS, and MD, and implemented by MD. PRB provided input into the spatial analysis of genotypes. The manuscript was written by RRK, RJO, MD, and LCMS with contributions from DMG. All authors discussed the interpretation of results and commented on the manuscript.

## Funding

RRK was funded by Wellcome Trust Senior Research Fellowship (081696/Z/06/A), RJO by Defra Project SE3243, PB by Scottish Government Project CR/(2009)/47, MD by Scottish Government as part of EPIC: Scotland’s Centre of Expertise on Animal Disease Outbreaks, and LCMS by Defra Project SE3285.

## Footnotes

2 Response to EIRs request for information about the costs of controlling BTB in 2013. FOI release. Bovine TB control costs in 2013. https://www.gov.uk/government/publications/bovine-tb-control-costs-in-2013.
