# Supplementary material for: Identifying genotype specific elevated-risk areas and associated herd risk factors for bovine tuberculosis spread in British cattle
Source: Epidemics. 2018 Sep;24:34–42. doi: 10.1016/j.epidem.2018.02.004 (PMC6105618; doi:10.1016/j.epidem.2018.02.004)
Supplement: Supplementary file 1 [file mmc1.docx]

**Supplementary Information:** **Identifying genotype specific elevated-risk areas and associated herd risk factors for bovine tuberculosis spread in British cattle**

R.J. Orton*, M. Deason^*^, P.R. Bessell, D.M. Green, R.R. Kao, L.C.M. Salvador

*joint first author

**
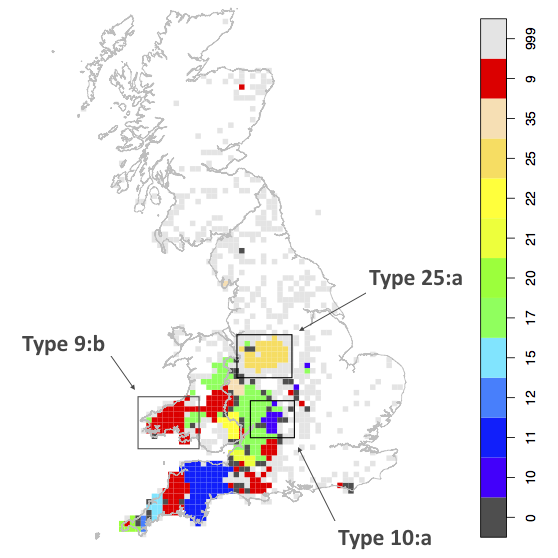
**

Figure S1. Dominant *Mycobacterium Bovis* spoligotypes in cattle by quadrat for all GB, based on all available data to 2010. Quadrats where more than 50% of typed isolates belong to an individual spoligotype are indicated by colour, with category “999” indicating insufficient data (<6 breakdowns) and "0" (dark grey) representing inconclusives - ie greater than 5 breakdowns but no one majority spoligotype identified, showing that most quadrats can be resolved to a single dominant type. Types 9:b, 10:a and 25:a are indicated by rectangular boxes.
