# Supplementary material for: Identifying genotype specific elevated-risk areas and associated herd risk factors for bovine tuberculosis spread in British cattle
Source: Epidemics. 2018 Sep;24:34–42. doi: 10.1016/j.epidem.2018.02.004 (PMC6105618; doi:10.1016/j.epidem.2018.02.004)
Supplement: Supplementary file 2 [file mmc2.docx]

**
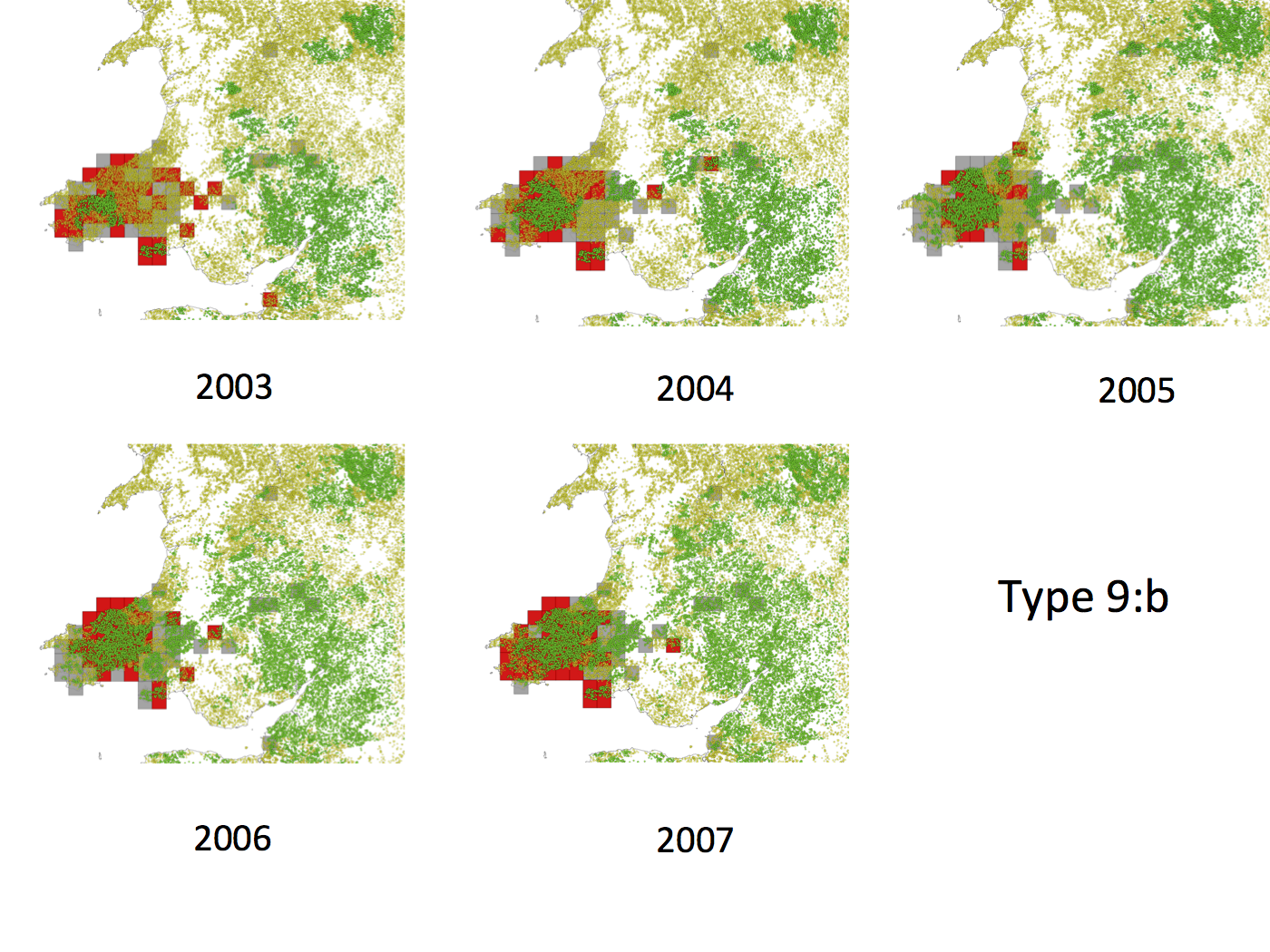
**

Figure S2. Attribution of Elevated-Risk Areas for Genotype 9:b, covering all years from 2003 to 2007. In red, quadrats deemed at elevated-risk (ER) for that year; in grey, non-ER quadrats. Herds under annual testing in green, herds under less frequent testing (typically quadrennial) in yellow.
